# Supplementary material for: Diversity, Composition, and Ecological Function of Endophytic Fungal Communities Associated with Erigeron breviscapus in China
Source: Microorganisms. 2025 May 6;13(5):1080. doi: 10.3390/microorganisms13051080 (PMC12113810; doi:10.3390/microorganisms13051080)
Supplement: Supplementary file 1 [file microorganisms-13-01080-s001.zip › microorganisms-3598427-supplementary.pdf]

Supplement Materials

Diversity, Composition and ecological function of endophytic fungal communities associated with *Erigeron breviscapus* in China

Yi Zhao[1], Hui Wu[2], Fang Wang[1], Liangzhou Zhao[3], Weijun Gong[3], Haiyan Li[3]*

[1]Key Laboratory of Chemistry in Ethnic Medicinal Resources, Yunnan Minzu University, Kunming 650500, China; febflower-zy@163.com (Y.Z.); wangfang@ymu.edu.cn (F.W.).

[2]Medical School, Shanghai Jiao Tong University, Shanghai 200025, China; hwu@shsmu.edu.cn (H.W.).

[3]Medical School, Kunming University of Science and Technology, Kunming 650500, China; zhaoliangzhou2021@163.com (L.Z.); weijung98@126.com (W.G.);*Correspondence: lhyxrn@163.com; Tel.: +86 (871)5920751.

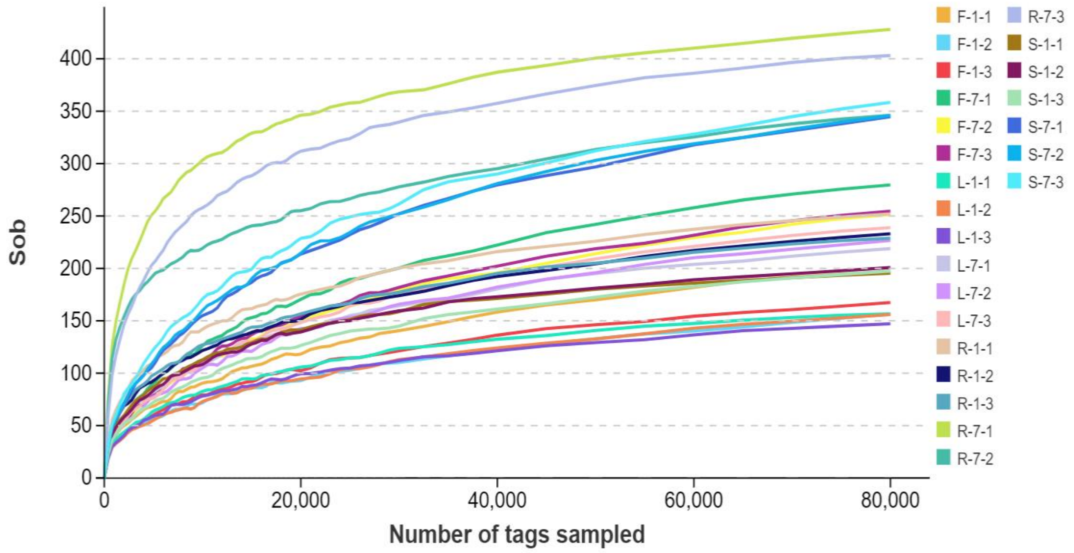

Figure S1 The rarefaction curve of endophytic fungal communities in *E. breviscapus* based on Sob index. F, L, R and S represent flowers, leaves, roots and stems, respectively. 1 and 7 stand for the sampling time January and July. Each group consists of 3 replicates. Sob represents the number of species observed.

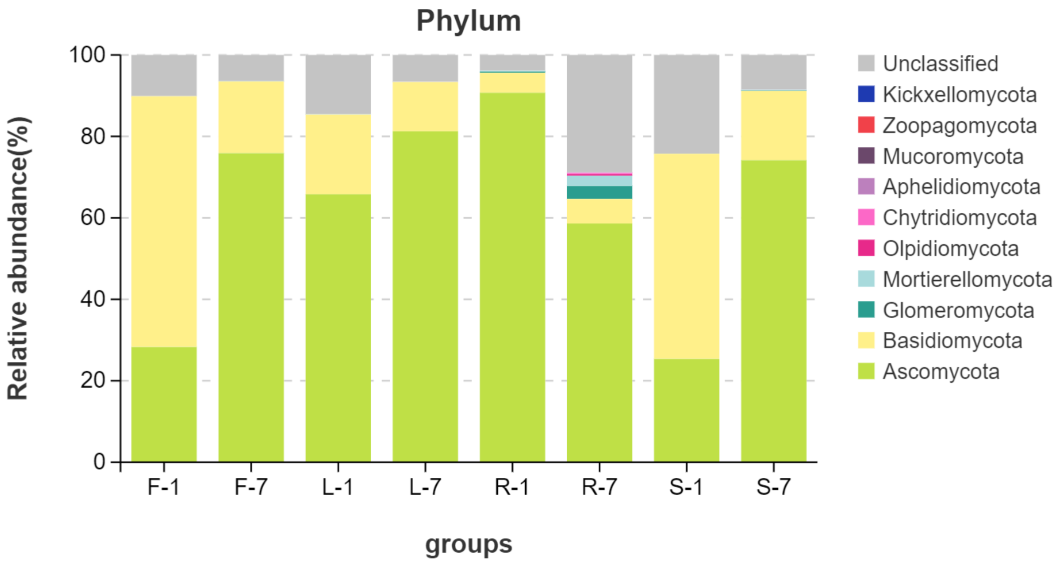

(a)

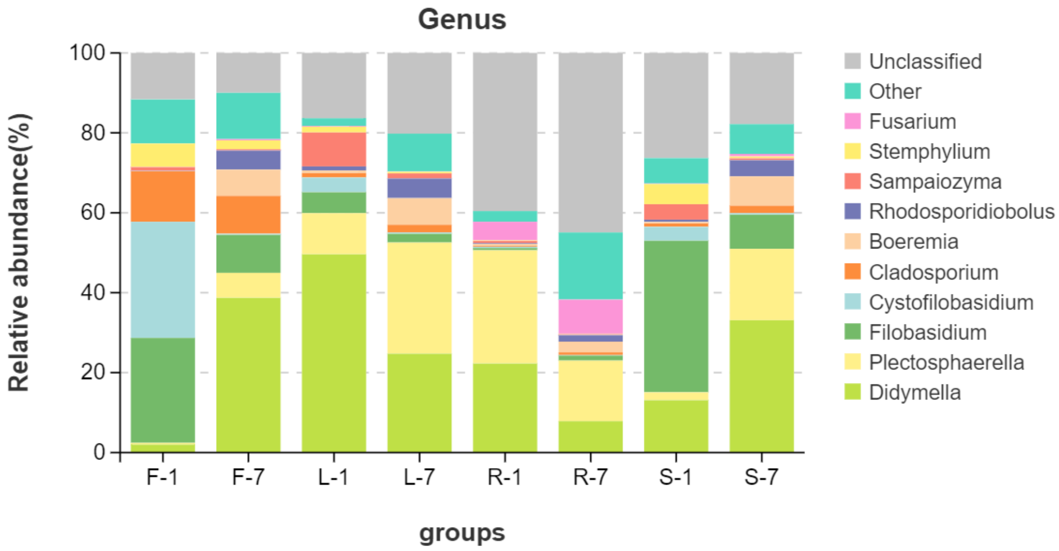

(b)

Figure S2 The relative abundance of endophytic fungi of *E. breviscapus* at (a) phylum and (b) genus levels. The box plot shows the top 10 detected species. Other species that can be classified were all assigned to “Other”. F, L, R and S represent flowers, leaves, roots and stems, respectively. 1 and 7 stand for the sampling time January and July. Each group consists of 3 replicates.

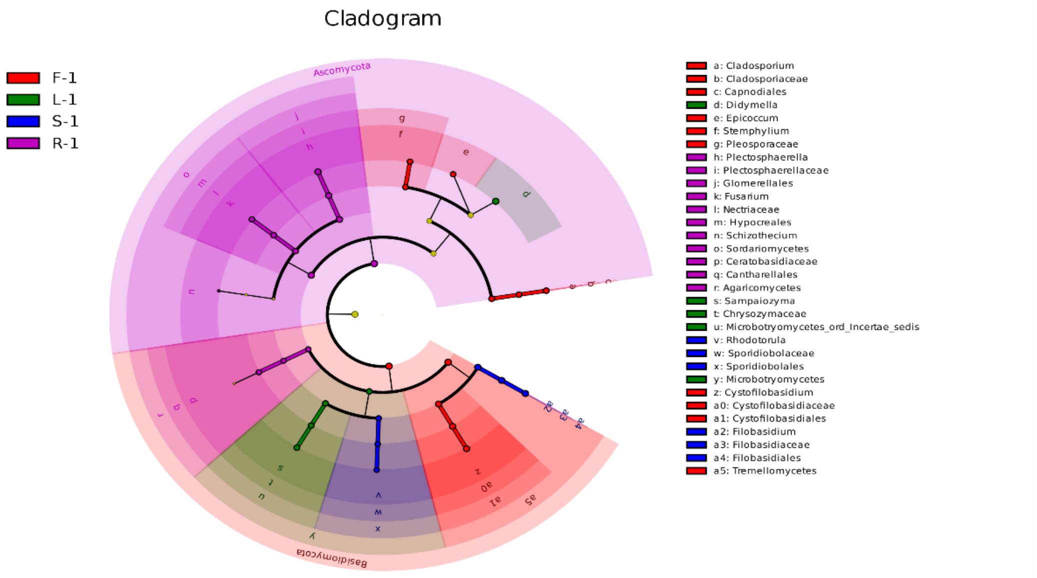

(a)

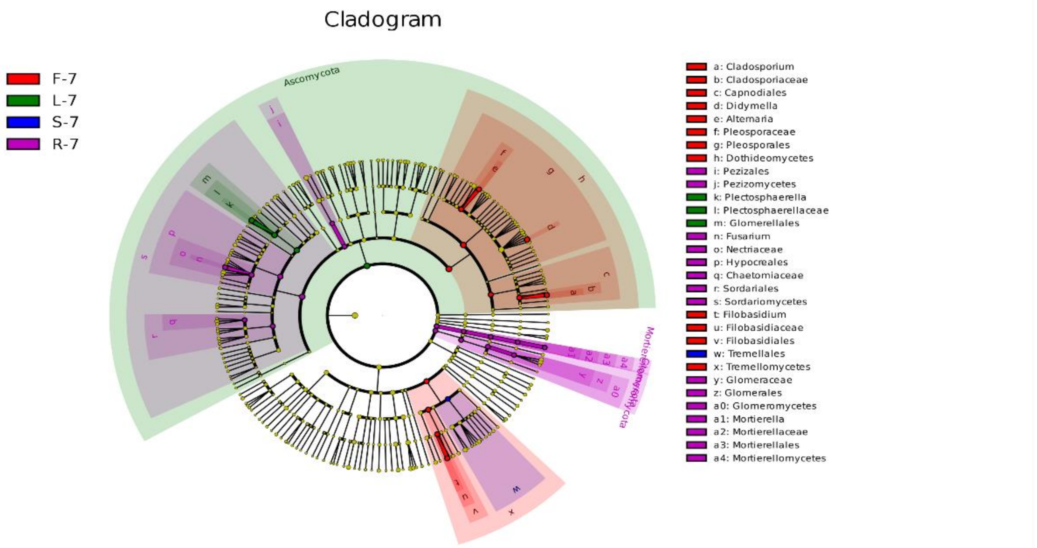

(b)

Figure S3 Linear discriminant analysis effect size (Lefse) analysis of differentially abundant taxonomic clades in fungal communities from *E. breviscapus*. LEfSe were shown in cladogram. (a) Samples were collected in January and analyzed by LEfSe. (b) Samples were collected in July and analyzed by LEfSe. All listed taxa were significantly enriched in their respective groups (Kruskal–Wallis test, $p < 0.05$, LDA score >4). Circle sizes in the cladogram plot are proportional to fungal abundance. F, L, R and S represent flowers, leaves, roots and stems, respectively. 1 and 7 stand for the sampling time January and July.

Table S1 The detailed information of tags and quality evaluation of the sequencing data.

Time	Sample Name	Raw Reads	Clean Reads	Raw Tags	Clean Tags	Effective Tags	Q20 (%)	GC (%)
January	F-1-1	134496	134186	118109	117420	117299	94.24	44.89
	F-1-2	129738	129458	115722	115074	115003	94.67	44.01
	F-1-3	132601	132295	120541	119913	119761	95.36	44.54
	L-1-1	120257	119878	101058	100416	100340	92.98	47.15
	L-1-2	132481	132238	120517	120070	120006	95.24	47.22
	L-1-3	136268	135847	115914	115270	115208	93.32	46.95
	R-1-1	120539	120394	113183	112618	112489	96.29	46.89
	R-1-2	137132	136919	129026	128513	128456	96.35	46.17
	R-1-3	121651	121448	114792	114204	114046	96.24	45.19
	S-1-1	126281	125581	103602	102872	102776	92.05	45.67
	S-1-2	136002	135493	118249	117535	117477	93.76	43.90
	S-1-3	126353	125634	99538	98781	98728	90.98	45.72
July	F-7-1	131032	130297	108055	107548	107446	94.07	46.46
	F-7-2	124753	124130	108420	107947	107593	94.93	45.83
	F-7-3	122795	121962	97134	96650	96440	93.40	47.11
	L-7-1	129276	128494	107240	106754	106609	94.11	47.29
	L-7-2	137950	136958	107023	106482	106339	93.07	47.78
	L-7-3	136725	135976	119269	118816	118628	95.01	46.56
	R-7-1	121114	118935	76651	75803	75738	90.49	48.71
	R-7-2	120979	118280	84940	83999	83919	92.17	47.82
	R-7-3	128476	126094	95412	94630	94512	92.79	47.46
	S-7-1	121955	120946	100991	100544	100450	94.03	46.55
	S-7-2	137940	135893	99231	98623	98523	91.95	47.70
	S-7-3	120382	119133	100862	100435	100368	94.35	46.29

F, L, R and S represent flowers, leaves, roots and stems, respectively; 1 and 7 stand for the sampling time January and July; 1, 2 and 3 represent triplicate samples.

TABLE S2 The Shannon (*H'*) indexes of endophytic fungi of *E. breviscapus*.

Sampling time	Shannon index (*H'*)			
	Flower	Leaf	Stem	Root
January	2.88 ±0.14a	2.52 ±0.09a	2.91 ±0.14a	3.14 ±0.20a
July	3.34 ±0.03a	3.48 ±0.01a	3.55 ±0.05a	5.40 ±0.22b

Data shown with Means ± standard error (n = 3). One-way ANOVA was used to analyze data from January. Kruskal–Wallis test was used to analyze data from July. Different letters in the same column indicated a significant difference at $p < 0.05$.

TABLE S3 The Simpson indexes of endophytic fungi of *E. breviscapus*.

Sampling time	Simpson			
	Flower	Leaf	Stem	Root
January	0.77 ±0.03a	0.70 ±0.02a	0.77 ±0.02a	0.76 ±0.04a
July	0.81 ±0.01a	0.84 ±0.01a	0.83 ±0.01a	0.94 ±0.01b

Data shown with Means ± standard error (n = 3). One-way ANOVA was used to analyze data from January and July. Different letters in the same column represented a significant difference ($p < 0.05$) was assessed by one-way ANOVA followed by LSD test.

TABLE S4 The Chao1 indexes of endophytic fungi of *E. breviscapus*.

Sampling time	Chao1			
	Flower	Leaf	Stem	Root
January	212.03 ±16.20a	182.71 ±4.97b	218.84 ±7.95a	266.06 ±1.53c
July	344.63 ±13.33a	275.17 ±10.45b	473.52 ±20.94c	464.69 ±17.39c

Data shown with Means ± standard error (n = 3). One-way ANOVA was used to analyze data from January and July. Different letters in the same column represented a significant difference ($p < 0.05$) was assessed by one-way ANOVA followed by LSD test.

TABLE S5 The ACE indexes of endophytic fungi of *E. breviscapus*.

Sampling time	ACE			
	Flower	Leaf	Stem	Root
January	219.49 ±16.27ab	187.41 ±8.33a	219.28 ±8.22ab	272.76 ±1.62b
July	346.49 ±13.17a	285.47 ±11.89b	450.08 ±10.89c	471.55 ±12.89c

Data shown with Means ± standard error (n = 3). Kruskal–Wallis test was used to analyze data from January. One-way ANOVA was used to analyze data from July. Different letters in the same column represented a significant difference ($p < 0.05$) was assessed by one-way ANOVA followed by LSD test.
